# The Association between Coagulation and Atrial Fibrillation

**DOI:** 10.3390/biomedicines12020274

**Published:** 2024-01-25

**Authors:** Saira Rafaqat, Sanja Gluscevic, Dimitrios Patoulias, Saima Sharif, Aleksandra Klisic

**Affiliations:** 1 Department of Zoology (Molecular Physiology), Lahore College for Women University, Lahore 54600, Punjab, Pakistan; 2 Department of Neurology, Clinical Center of Montenegro, 81000 Podgorica, Montenegro; 3 Outpatient Department of Cardiometabolic Medicine, Second Department of Cardiology, Aristotle University of Thessaloniki, General Hospital “Hippokration”, 54642 Thessaloniki, Greece; 4 Faculty of Medicine, University of Montenegro, 81000 Podgorica, Montenegro; 5 Center for Laboratory Diagnostics, Primary Health Care Center, 81000 Podgorica, Montenegro

**Keywords:** atrial fibrillation, coagulation, coagulation factors, coagulation markers, pathogenesis

## Abstract

The existing literature highlights the presence of numerous coagulation factors and markers. Elevated levels of coagulation factors are associated with both existing and newly diagnosed cases of atrial fibrillation (AF). However, this article summarizes the role of coagulation in the pathogenesis of AF, which includes fibrinogen and fibrin, prothrombin, thrombomodulin, soluble urokinase plasminogen activator receptor, von Willebrand factor, P-selectin, D-dimer, plasminogen activator inhibitor-1, and platelet activation. Coagulation irregularities play a significant role in the pathogenesis of AF.

## 1. Introduction

Atrial fibrillation (AF) is responsible for creating an irregular and unpredictable heart rhythm. It is the prevailing form of sustained arrhythmia, characterized by the disorganized, rapid and irregular activation of the atria, and it leads to an irregular response in the ventricles. These effects result in the loss of atrial contractility, preventing the complete emptying of blood from the atrial appendage. Consequently, there is an increased risk of clot formation and subsequent thromboembolic events. Depending on its specific characteristics and duration, AF can be classified into various subtypes [1]. The process of blood coagulation follows a sequential series of events triggered by the activation of either the contact-activation pathway or the tissue-factor pathway, which eventually converge to form a common pathway. The result of this process is the formation of fibrin, which serves as a key component of a blood clot (thrombus). Coagulation techniques involve the utilization of fresh non-anticoagulated whole blood, anticoagulated whole blood (treated with citrate), or platelet-rich plasma [2].

There is a substantial amount of evidence suggesting the presence of a prothrombotic or hypercoagulable state in AF. However, the causes contributing to this state are complex and cannot be solely attributed to the stagnation of blood flow. In AF, various abnormal changes occur in the atrial wall (both anatomical and structural, referred to as “vessel wall abnormalities”), in addition to the presence of spontaneous echo contrast indicating abnormal flow patterns and stagnation (“flow abnormalities”). Additionally, there are abnormal changes in coagulation, platelet function, and other pathophysiological pathways (“abnormalities of blood constituents”), all of which have been extensively studied and documented [3,4,5].

Elevated levels of coagulation factors are associated with both existing and newly diagnosed cases of AF. The strength of these associations becomes more pronounced in studies with a higher prevalence of AF. Long-term research provides some evidence suggesting that the development of AF may be influenced by a prothrombotic state. These findings support the notion that both prothrombotic and hypofibrinolytic conditions underlie the occurrence of AF and further contribute to its progression by promoting additional structural changes in the atria [6].

The adverse thromboembolic consequences of AF can be attributed to multiple factors beyond the hemodynamic changes caused by this arrhythmia. These factors encompass structural heart disease, inappropriate or excessive platelet activation, disrupted hemostasis in conjunction with abnormal blood flow, and impairment of the clotting cascade. Virchow’s triad, which encompasses abnormal blood flow, irregular vascular structure, and anomalous blood constituents, can help explain the increased likelihood of thrombus formation (thrombogenesis) in AF [7].

The research indicates that AF leads to enhanced platelet aggregation and coagulation, with the duration of AF playing a role in regulating these processes. Within 12 h of AF onset, there is an acceleration in platelet activity and coagulability [8]. Furthermore, in AF patients, the left atrial appendage (LAA) forms denser fibrin clots compared to the peripheral blood, which could contribute to the development of LAA thrombi and thrombi related to devices [9].

Numerous coagulation biomarkers have been presented in the literature [6,10,11,12]. However, this review article represents a summary of the role of coagulation in the pathogenesis of AF.

## 2. Major Coagulation Factors and Markers in Atrial Fibrillation

**Figure 1:**
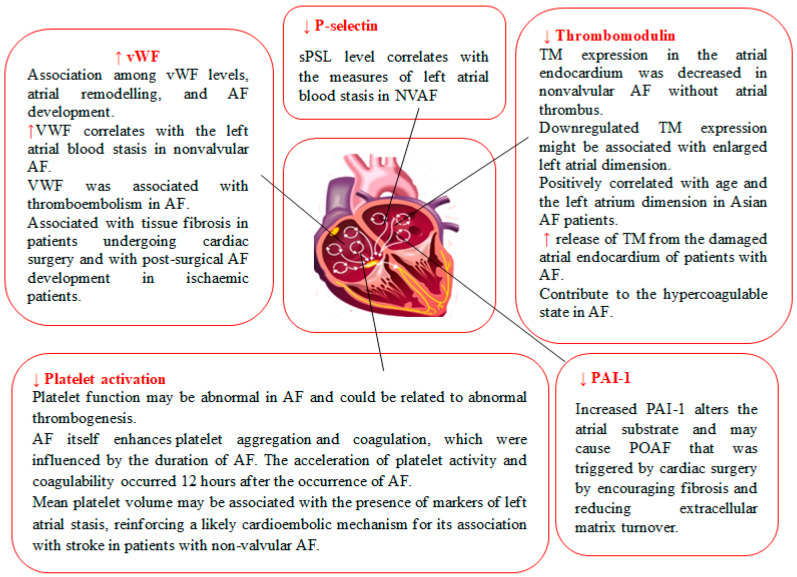
Circulatory levels and pathophysiological aspects of coagulation factors and markers in atrial fibrillation. Legend: increased circulatory levels of coagulation markers and activators (↑), decreased circulatory levels of coagulation markers and activators (↓), atrial fibrillation (AF), von Willebrand factor (vWF), thrombomodulin (TM), plasma soluble P-selectin (sPSL), plasminogen activator inhibitor-1 (PAI-1), postoperative atrial fibrillation (POAF), nonvalvular atrial fibrillation (NVAF).

Coagulation factors are responsible for the formation of blood clots, which are vital for stopping bleeding after injury or trauma. When blood vessels are damaged, coagulation factors work together in a coordinated manner to form a stable clot at the site of the injury. This clot seals the wound, preventing further blood loss. Coagulation factors help prevent excessive bleeding by promoting the formation of a stable clot. In individuals with deficiencies or abnormalities in coagulation factors, the blood may not clot properly, leading to prolonged bleeding or spontaneous bleeding, even without an injury. Disorders such as hemophilia are characterized by deficiencies in specific coagulation factors and require specific treatments to control bleeding. There are numerous coagulation factors and markers. However, this article only focuses on fibrinogen and fibrin, prothrombin, thrombomodulin, soluble urokinase plasminogen activator receptor, von Willebrand factor, P-selectin, D-dimer, PAI-1, and platelet activation in AF, as depicted in Figure 1.

## 3. Fibrinogen and Fibrin

The fibrinogen molecule is a glycoprotein consisting of two sets of polypeptide chains: 2Aα, 2Bβ, and 2γ. It weighs about 340 kilodaltons (kDa) and is formed by connecting these chains through 29 disulfide bridges. The synthesis of fibrinogen mainly occurs in hepatocytes. The assembly process involves a stepwise progression, in which individual chains first combine to form Aα-γ and Bβ-γ complexes. These complexes then further assemble to create Aα/Bβ/γ half-molecules. Finally, the half-molecules come together to form hexametric complexes known as (Aα/Bβ/γ)_2_ [13]. Fibrinogen is a crucial coagulation factor and the primary protein found in the plasma. When plasma fibrinogen levels are low, it can lead to a higher susceptibility to bleeding, due to impaired primary and secondary hemostasis. Additionally, fibrinogen is traditionally considered a positive acute-phase reactant protein and serves as an independent indicator for predicting coronary heart disease events. This evaluation examines the various methods available for measuring fibrinogen levels and provides recommendations regarding their appropriate application [14].

Higher plasma fibrinogen and fibrin D-dimer levels are linked to AF. These markers are present in moderate levels in PAF patients, which is consistent with their intermediate risk of thromboembolism [14,15]. The proximity of many locations crucial for factor XIIIa-dependent cross-linking to the α-fibrinogen Thr312Ala polymorphism increases the likelihood that it has an impact on fibrin-clot stability. Carter et al. concluded that no correlation between Thr312Ala and stroke or a stroke subtype was found. Regarding poststroke mortality, however, Thr312Ala and AF did significantly interact. These findings may have significant repercussions for identifying those most at risk of having thromboembolic consequences, since they suggest that the Thr312Ala polymorphism may enhance susceptibility for intra-atrial clot embolization [16]. Also, there were no significant changes in plasma fibrinogen with cardioversion in either group of patients [17].

Increased fibrinogen levels were prospectively correlated with a higher risk of AF, which supports the notion that inflammation plays a role in the genesis of AF [18]. In contrast to non-AF controls, none of the examined hemostasis or fibrinolysis metrics revealed any discernible intracardiac changes in AF patients. On both the intracardiac and systemic levels, AF patients have higher FVIII and vWF levels, most likely as a result of endothelial injury [19].

Compelling evidence suggests that patients with AF, even those in sinus rhythm following paroxysmal and persistent AF, experience a hypercoagulable state. This is characterized by heightened thrombin generation and increased formation of fibrin, as indicated by elevated levels of soluble fibrin monomers and D-dimer. Notably, patients with AF have been shown to develop a denser fibrin meshwork which is relatively resistant to plasmin-mediated breakdown. The presence of stroke risk factors in AF, such as diabetes, heart failure, hypertension, previous myocardial infarction, stroke, and advanced age, has been associated with clot characteristics that promote thrombosis, including reduced clot permeability and susceptibility to lysis. Important biomarkers such as cardiac troponins and N-terminal pro-brain natriuretic peptides were correlated with thrombin generation and markers related to fibrin in AF patients. Recently, studies have demonstrated that increased fibrin-clot density (measured as low clot-permeability in plasma-based assays) and impaired fibrinolysis, assessed while the patient was not receiving anticoagulation, might predict ischemic cerebrovascular events in patients with AF who were being treated with vitamin K antagonists or rivaroxaban [20].

Increased formation of compact fiber networks has been observed in cases of AF and ischemic stroke. In a longitudinal cohort study aiming to assess whether the density of fibrin clots could serve as an indicator for predicting the risk of thromboembolism and bleeding in patients with atrial fibrillation who were receiving vitamin K antagonists, it was demonstrated that the presence of undesirable fibrin characteristics, characterized by the formation of denser fibrin networks, plays a role in determining the effectiveness and safety of anticoagulation therapy using vitamin K antagonists for individuals with AF [21].

The precise mechanisms through which chronic kidney disease (CKD) affects fibrin-clot characteristics in AF are not well understood. Significant alterations in fibrin-clot properties associated with both AF and CKD have been found, which could potentially explain the elevated risk of thromboembolic complications. However, these changes in fibrin-clot properties were not found to be caused by modifications in the distribution of lipoproteins [22].

## 4. Prothrombin

Thrombin is a serine protease encoded by the F2 gene in humans. During the clotting process, prothrombin (coagulation factor II) undergoes proteolytic cleavage to form thrombin. Thrombin, acting as a serine protease, then plays a vital role by converting soluble fibrinogen into insoluble fibrin strands. Additionally, thrombin catalyzes various other reactions associated with coagulation [23,24].

Important blood coagulation regulation is provided by the protein C system, which controls the activities of factor VIIIa (FVIIIa) and factor Va (FVa), cofactors in the activation of factor X and prothrombin, respectively. The system consists of circulating and membrane-bound proteins that aggregate onto cell surfaces to form multimolecular complexes. A vital component of the system, vitamin-K-dependent protein C, circulates in blood as zymogen to a serine protease that acts as an anticoagulant. On the surface of endothelial cells, thrombin coupled to thrombomodulin, a membrane protein, effectively activates it. Further inducing protein C activation is the endothelial protein C receptor (EPCR). By destroying FVIIIa and FVa on the surface of negatively charged phospholipid membranes, activated protein C (APC) and its cofactor protein S prevent coagulation. In addition to protein S, intact FV—which, like thrombin, is a Janus-faced protein with procoagulant and anticoagulant properties—is necessary for the efficient destruction of FVIIIa by APC. APC binds to EPCR and proteolytically cleaves protease-activated receptor 1 (PAR-1), exhibiting anticoagulant, anti-inflammatory, and anti-apoptotic effects. The most frequent causes of venous thrombosis are genetic abnormalities affecting the protein C system, which is physiologically significant. The three-dimensional structures of some of the proteins in the protein C system are known, and the proteins are made up of numerous domains. We are learning more and more about this complex and intriguing atomic-level molecular situation as the molecular recognition of the protein C system is gradually being uncovered [25]. The primary inhibitor of thrombin is antithrombin (AT), formerly known as AT III. This inhibitor of serine proteases binds to thrombin, factor IXa, Xa, XIa, and XIIa, deactivating them. Heparin increases the enzymatic activity of AT. Nonetheless, heparin’s modest plasma concentration has no effect on the in vivo activation of AT. Heparin sulphate that is found on the surface of endothelial cells binds to AT, activating it. Reticuloendothelial cells extract the complex formed when AT binds coagulation factors in a 1:1 ratio. Additionally, α1-antitrypsin, α2 macroglobulin, and heparin cofactor II are further thrombin inhibitors [26,27].

According to the research, it is critical to develop biomarkers that show how strongly an anticoagulant is being used in connection with bleeding or thrombotic events. To determine, for example, if the non-specific thrombin production marker prothrombin activation fragments 1 and 2 (F1 + 2) change following the administration of an FXa inhibitor to AF patients. In the peri-ablation research, higher F1 + 2 levels were linked to a bigger fall in C-Xa levels following periprocedural disconnecting of FXa inhibitors in the rivaroxaban group, compared to the apixaban group. In individuals with AF, F1 + 2 showed a small and inverse correlation with plasma concentrations of apixaban and rivaroxaban. It would have been necessary to conduct a larger investigation to determine whether persistent thrombin production after anticoagulation was linked to an increased risk of clinical events [28].

Patients with AF frequently utilize rivaroxaban, a direct factor Xa inhibitor, to lower their risk of stroke. It is unclear why rivaroxaban medication in individuals with AF does not correlate with the prothrombin time (PT) of the international normalized ratio (INR). In conclusion, several factors influence the PT-INR of the INR in patients with AF on rivaroxaban. Clinical results for AF patients using rivaroxaban who have varying INR levels may be comparable [29].

According to an investigation based on the amount of time that passed between taking rivaroxaban and drawing blood, PT/INR is most affected up to 12 h after taking the medication, before returning to normal levels in the hours that follow and before the next dosage. In contrast to warfarin, it was concluded that for appropriately interpreting a laboratory test to evaluate hemostasis, notably PT and its derivatives, blood must be drawn from patients using rivaroxaban, and that this time interval must be known [30].

Although neither PT-INR nor aPTT were carried out for patients with AF receiving direct oral anticoagulants (DOACs) in randomized trials, these tests were frequently utilized and well known to clinical doctors. Among AF patients using rivaroxaban or dabigatran, respectively, Chao et al. determined whether there was a correlation between PT-INR or aPTT ratio and the risks of ischemic stroke/systemic embolism (IS/SE) and serious bleeding. The incidence of bleeding episodes for rivaroxaban or dabigatran in Asian AF patients was not related to PT-INR or aPTT ratios. There was a decreased risk of IS/SE in patients on rivaroxaban when their INR was less than 1.5. Patients receiving rivaroxaban with an INR less than 1.5 should have the appropriate doses of DOACs and their compliance verified [31]. A Japanese study showed that a prolonged peak prothrombin time (≥20 s) is indicative of a higher risk of bleeding in patients with non-valvular AF using rivaroxaban, and both trough and peak plasma soluble fibrin (SF) levels were lower, as compared with the baseline. Regardless of previous anticoagulant use, PT and SF were also significant indicators of coagulation status in rivaroxaban-treated subjects [32].

## 5. Thrombomodulin

Thrombomodulin is a crucial factor in maintaining a proper balance of coagulation. When present in endothelial cells, it forms a complex with thrombin that alters the enzyme’s substrate specificity, acting as an intrinsic inhibitor. Thrombin bound to thrombomodulin is unable to activate protein C, which has a strong anticoagulant effect [33]. Despite its importance, few studies investigate thrombomodulin expression in the atrial endocardium of patients with AF. In patients with nonvalvular atrial fibrillation (NVAF) and no atrial thrombus, the expression of thrombomodulin in the atrial endocardium is reduced, whereas tissue-factor pathway inhibitor expression is not affected. This decrease in thrombomodulin expression may be linked to an enlarged left-atrial dimension [34].

Thrombomodulin has a crucial function in controlling blood clot formation on the endothelial cell surface. When thrombin binds to thrombomodulin, it triggers the activation of protein C, leading to a series of events that prevent blood from clotting and reduce inflammation in the blood vessels [35]. At baseline, the restored sinus rhythm group had a higher concentration of soluble thrombomodulin (s-TM) than the group with recurrent AF. After 6 months of catheter ablation, both s-TM and plasminogen activator inhibitor-1 (PAI-1) levels had increased [36].

Another study highlights endothelial dysfunction mediated by sTM, which is a well-known marker of endothelial damage. Specifically, sTM is a membrane protein that regulates thrombin activity on the endothelial surface and activates the anticoagulant protein C. Elevated levels of sTM in plasma have been linked to the release of the protein from damaged endothelial cells [37]. Such elevated levels of sTM in plasma could potentially increase the likelihood of thromboembolic events, due to the endothelial surface’s conditioning for a procoagulant state. In this report, elevated levels of sTM were significantly associated with persistent AF during long-term follow-up. An association has been suggested between the long-term maintenance or recurrence of AF and inflammatory or endothelial parameters, such as sTM and C-reactive protein (CRP), respectively [38].

AF is a known cause of thromboembolism. While reduced blood flow and a hypercoagulable state are considered the primary mechanisms, a dysfunctional endocardium may also play a role in thrombogenesis. Rapid atrial pacing can acutely decrease the expression of genes for thrombomodulin (TM) and tissue-factor pathway inhibitor (TFPI) in the endocardium, causing an imbalance in local coagulation on the internal surface of the atrial cavity. It has been confirmed that endocardial dysfunction plays a role in AF. Normally, the atrial endocardium acts as a barrier to prevent blood from clotting during sinus rhythm. Immunohistochemical analysis showed that the surface of the endocardium was almost completely covered with anticoagulant molecules such as TM and TFPI. However, several hours of rapid atrial excitation caused a decrease in their gene expression, which appeared to remove these essential anticoagulant barriers. These findings suggest the potential usefulness of supplementing anticoagulant molecules deficient in AF [39].

A retrospective analysis was conducted on data from NVAF patients who underwent minimally invasive surgical AF ablation at a specific center. The expression of von Willebrand factor (vWF) and thrombomodulin in the atrial endocardium and their relationships with the rhythm outcomes following the procedure were investigated. The results suggest that the expression of vWF and TM in the atrial endocardium might be linked to the recurrence of AF after minimally invasive surgical AF ablation. Specifically, patients who experienced AF recurrence had higher vWF expression and lower TM expression [40]. Patients with AF had significantly lower soluble thrombomodulin levels compared to control subjects [41].

Different factors, among them inflammation, endothelial dysfunction, coagulation system’s dysfunction, and activation of platelets, have been shown to be contributors to thromboembolism in AF patients. The link between serum endothelial function biomarkers and known thromboembolic risk factors in a Korean population with permanent and persistent AF found a strong and consistent correlation between endothelial function markers and thromboembolic risk factors. Moreover, thrombomodulin was elevated in older patients with a history of thromboembolism and left-ventricular dysfunction, and positively correlated with age and left-atrial dimension (LAD). The importance of endothelial dysfunction in the development of thromboembolism in Asian AF patients was highlighted to suggest that efforts to improve endothelial function should be considered to reduce the risk of thromboembolism [42].

AF is the most common type of irregular heart rhythm and is associated with a five-fold increase in the risk of stroke, compared to people with normal sinus rhythm. Additionally, sTM is a marker of endothelial dysfunction and may contribute to the increased risk of blood clots in people with AF. The level of sTM in the plasma of patients with persistent AF before and after restoration of sinus rhythm following direct current cardioversion (CV) was examined. Patients with persistent AF and normal left-ventricular systolic function had lower levels of plasma sTM, compared to those with normal sinus rhythm, likely due to the use of chronic oral anticoagulant therapy in the AF group. It was found that restoration of sinus rhythm through CV did not significantly affect sTM plasma levels when measured 24 h after the procedure [43].

## 6. Soluble Urokinase Plasminogen Activator Receptor

Soluble urokinase plasminogen activator receptor (suPAR) is a non-specific parameter of inflammation. It is a soluble form of the urokinase receptor. The urokinase cleaves plasminogen to plasmin, which stimulates the fibrinolysis cascade for dissolving thrombi. The receptor suPAR is thought to have a chemotactic properties, enabling migration of leukocytes. The active form of the receptor is found on different cells, such as tissue macrophages, white blood cells, endothelial cells, fibroblasts, renal tubular cells, and cardiomyocytes. It can be determined in plasma, saliva, and urine [44,45].

Accordingly, suPAR is a soluble form of the urokinase plasminogen activator receptor found on cell membranes that plays a role in converting plasminogen to plasmin, which can initiate a process leading to thrombosis or degradation of the extracellular matrix. Elevated levels of suPAR in the circulation indicate increased activation of the immune system, and it has been identified as a new biomarker for chronic low-grade inflammation. In addition, suPAR has been linked to various medical conditions and can be more effective than traditional inflammation markers, such as CRP, in predicting the risk of cardiovascular disease [46].

Circulating suPAR, a biomarker of immune activation and low-grade inflammation, could potentially serve as a novel indicator of cardiovascular disease. In patients with cardiac conditions, suPAR levels were linked to AF, especially non-paroxysmal AF (NPAF), and statistical analysis found that the association between suPAR and NPAF was not affected by age, gender, systolic blood pressure, estimated glomerular filtration rate, CRP, or plasma brain-type natriuretic peptide (BNP). However, additional research is needed to determine whether elevated suPAR plays a role in the development of AF [47].

Moreover, suPAR is a new marker of inflammation that is released from neutrophils, T cells, and macrophages. Unlike other inflammatory markers, which are mainly acute-phase proteins produced in the liver, suPAR has been linked to heart failure (HF) and AF. The relationship between suPAR and incident HF and AF in a population-based cohort was examined. In this study, suPAR was associated with both increased levels of NT-proBNP in plasma and a higher incidence of HF, but not with AF, in middle-aged individuals [48]. Also, another study showed that suPAR levels were associated with low LVEF and high BNP, but not with left-ventricular hypertrophy [49].

Another study aimed to investigate whether suPAR levels could predict new-onset AF in a large cohort of acute medical patients during long-term follow-up. The results showed that plasma suPAR levels were independently associated with new-onset AF in this population. However, the prediction of AF was not improved when suPAR was considered with other risk factors. A link between suPAR and consequent AF risk was found in a population that sought emergency care for disorders unrelated to AF, with a doubling of suPAR corresponding to a 20% increase in the risk of incident AF. Despite these findings, further research is needed to better understand the role of inflammation and inflammatory markers, including suPAR, in the development of AF [50].

## 7. Von Willebrand Factor

Von Willebrand factor (vWF) is a glycoprotein present in the plasma which plays a crucial role in maintaining hemostasis by encouraging platelet adhesion and aggregation at the location of vascular damage. When endothelial cells are activated or injured, there is an increase in the release of vWF. Higher levels of vWF in the plasma indicate endothelial dysfunction, which increases the likelihood of developing atherosclerosis and thrombosis [51].

vWF is essential for platelet adhesion and aggregation at high shear rates, especially in the presence of stenotic or ruptured atherosclerotic plaque lesions in coronary arteries. Several studies have investigated the link between vWF plasma levels and thromboembolic cardiovascular events, and while the association is weak in the general population, these levels are significantly predictive of adverse cardiac events in patients with preexisting vascular disease. During acute coronary syndrome, vWF levels typically increase, and the degree of increase is a strong predictor of negative outcomes. Also, vWF plays an active role in the development of myocardial infarction. Given its crucial role in thrombogenesis, vWF has become a promising target, encouraging the development of new antiplatelet therapies that specifically inhibit vWF [52].

HF and AF have similar mechanisms, both of which can contribute to a higher risk of blood clot formation and thrombosis. An increased level of vWF in the blood has been linked to a greater likelihood of thromboembolism and cardiovascular problems. The outcomes of prospective studies indicate that, in patients with non-valvular AF, high vWF plasma concentrations, in conjunction with established cardiovascular risk factors, might more accurately predict clinical outcomes, such as stroke or all-cause death. Further research is needed to investigate whether vWF plasma concentrations could be used to identify patients with a low CHA2DS2-VASc score who are at moderate risk of stroke or other cardiovascular events and might benefit from oral anticoagulant (OAC) therapy [53].

After cardiac surgery, AF is linked to a higher risk of illness and death, and vWF is regarded as an indicator of endothelial dysfunction or damage. Therefore, vWF levels could be a useful biomarker for predicting the occurrence of AF following cardiac surgery. Additionally, the question of whether there is a connection between vWF and tissue remodeling that could contribute to post-surgical AF has been investigated. It was found that, in patients who underwent cardiac surgery, elevated plasma vWF levels were associated with tissue fibrosis and the development of post-surgical AF in those with ischemia. The results indicate a correlation between vWF levels, atrial remodeling, and the onset of AF, as demonstrated by higher vWF expression in the right-atrial tissue of ischemic patients who developed post-surgical AF [54].

In patients with NVAF, an increase in vWF is linked to blood stasis in the left atrium. However, the long-term impact of elevated vWF in patients with NVAF is not clearly understood. Among patients with NVAF, those who experience complications related to thromboembolism (TE) have higher activity levels and concentrations of vWF antigen. Furthermore, vWF is an independent predictor of poor outcomes, i.e., death and a combination of death and TE, even after adjustment for echocardiographic and clinical parameters. Therefore, vWF can be reliable in identification of patients with a high level of risk, and enable additional stratification beyond the assessment of CHA2DS2-VASc [55].

In elderly patients with AF, an increase in baseline levels of vWF is significantly linked to a greater likelihood of experiencing major adverse cardiac events (MACEs) and death from any cause. This risk is particularly pronounced in patients with non-valvular AF. Nonetheless, additional well-designed prospective studies are necessary to validate these results [56]. Moreover, left-atrial blood stasis is linked with higher risk of left-atrial appendage thrombus (LAAT) and stroke in AF patients, while vWF is linked to thromboembolism in AF, and the thrombogenic activity of vWF is proportional to multimer size, which is regulated by vWF-cleaving protease (ADAMTS13). This supports a direct link between vWF and measures of whole-blood stasis in the left atrium. The association between vWF levels and the severity of left-atrial blood stasis can explain the propensity for thrombosis in AF patients. Elevated vWF may also help identify AF patients at risk for LAAT. Although there may be a temptation to establish a cut-off value for vWF levels that distinguishes stroke risk, this is an oversimplification of the complex process of stroke in AF. Similar paradigms have been proposed for hypertension and cholesterol, but despite being undisputed risk factors for coronary disease, their risk and treatment targets remain indistinct and are constantly evolving. The thrombosis in AF is similarly complex [57].

In the same way, the role of vWF as a marker of endothelial dysfunction in predicting prognosis was studied in anticoagulated AF patients. It was investigated whether adding vWF levels to clinical risk stratification schemes would improve the prediction of event risk. It was found that vWF can be used as a simple prognostic biomarker in anticoagulated AF patients. Even though adding vWF levels to the HAS-BLED and CHA2DS2-VASc and led to improvement in prediction for some endpoints, these changes had no significant clinical value on practical decision-making [58].

Another study suggested that patients with AF who received OAC treatment and had a history of stroke exhibited changes in their vWF levels, activity, and molecular structure. These modifications were linked to thrombotic complications, underscoring the potential role of vWF as a biomarker or therapeutic target. However, further studies with larger sample sizes are necessary to validate the hypothesis. The identification of a new risk factor may facilitate the development of novel preventive and treatment approaches [59].

## 8. P-selectin

P-selectin is an adhesion molecule that plays a crucial role in the interaction between blood cells and the endothelium. It is found on the external cell surface of activated endothelium cells and activated platelets, with most of the measured plasma P-selectin originating from platelets. P-selectin is involved in leukocyte and platelet adhesion to the endothelium, and animal studies have shown that it is also important in atherogenesis. Active atherosclerotic plaques express higher levels of P-selectin than do inactive, fibrotic plaques. Animals that lack P-selectin exhibit a lower tendency towards the formation of atherosclerotic plaques. Increased plasma soluble P-selectin levels have been recorded in many cardiovascular disorders, such as hypertension, coronary artery disease, and AF [60].

P-selectin is a crucial molecule that plays a role in cell adhesion and is found in the storage granules of platelets and the Weibel–Palade bodies of endothelial cells. When activated, P-selectin moves to the plasma membrane and facilitates interactions between neutrophils and platelets, as well as those between neutrophils and endothelial cells [61]. Patients with NVAF have elevated levels of P-selectin, a biomarker of platelet and endothelial cell activation. However, there is controversy regarding the relationship between sP-selectin levels and thromboembolic complications in these patients. It was presumed that plasma soluble P-selectin (sPSL) levels are linked with measures of left-atrial blood stasis in NVAF patients. Direct relationships between sPSL levels and severe blood stasis in the left atrium, as well as a clinically confirmed prothrombotic state (CHA2DS2-VASc score ≥ 1) and the presence of LAAT were demonstrated [62].

The occurrence of gene polymorphisms in the sP-selectin gene is linked to elevated serum levels of sP-selectin or a higher risk of thromboembolic events. This implies that individuals with nonvalvular AF and a history of thromboembolic events may have a relevant genetic predisposition [63]. Also, peripheral sP-selectin levels were associated with hypertension before ablation and with persistent AF after ablation [64].

AF patients have been shown to experience platelet activation and decreased levels of nitrite and nitrate (NOx), which are stable end products of nitric oxide (NO). In an animal study, the changes over time in plasma NOx levels and the expression of P-selectin on platelets after AF onset were explored, seeking to determine whether these biomarkers could be risk factors for silent cerebral infarction in AF patients. The irregular heart rate characteristic of AF reduced NO synthesis. It was found that the enhanced expression of P-selectin on platelets, which was linked with lower NO levels, was a risk factor for silent cerebral infarction in AF patients [65].

To understand the role of platelets in AF and their response to antithrombotic therapy, researchers developed a new method to measure the absolute amount of P-selectin per platelet (pP-selectin) using cell lysis. This allowed them to compare pP-selectin levels in AF patients with healthy controls and with established indices of platelet activation like plasma soluble P-selectin (sP-selectin) and β-thromboglobulin. The level of pP-selectin, which is a specific protein, was considerably reduced in AF patients who were not receiving any antithrombotic treatment, as compared to healthy individuals. However, the levels of sP-selectin and beta-thromboglobulin were not significantly different between the two groups, and these levels did not differ significantly in patients taking either aspirin or warfarin. Nonetheless, in AF patients, pP-selectin was lower in those taking aspirin compared to those taking warfarin. In healthy controls, there was a significant positive correlation between pP-selectin and sP-selectin levels. Conversely, in AF patients not receiving any antithrombotic therapy, there was an inverse correlation between pP-selectin and sP-selectin levels. Lower levels of pP-selectin in AF patients may indicate depletion after platelet activation. Aspirin was found to further decrease pP-selectin levels, compared to warfarin. it was possible to determine the amount of P-selectin per platelet using the principle of platelet lysis, and this might be regulated in the megakaryocyte through a cyclooxygenase-dependent pathway. The increased risk of stroke and thromboembolism in AF might be due to a prothrombotic or hypercoagulable state with abnormalities of hemostasis and platelet activation [66].

Lymphatic formation depends on C-type lectin-like receptor 2 (CLEC-2), which is strongly expressed on platelets and a subset of myeloid cells. It has been demonstrated that CLEC-2 promotes thrombus development at inflammatory locations but plays a small or inconsequential part in hemostasis. This suggests that CLEC-2, without causing hemostatic harm, is a possible therapeutic target in thrombo-inflammatory diseases [67].

It has been discovered that the platelet-activating snake venom rhodocytin is bound to CLEC-2. Like the collagen receptor glycoprotein (GP) VI/FcRγ-chain complex, CLEC-2 has the ability to trigger strong platelet activation signals when paired with Src, Syk kinases, and phospholipase Cγ2. In a manner different from GPVI/FcRγ, which triggers platelet activation via the immunoreceptor tyrosine-based activation motif (ITAM) with tandem YxxL motif, CLEC-2 communicates through the immunoreceptor hemi-ITAM. Podoplanin has been discovered to be the endogenous ligand of CLEC-2. It is expressed on the surface of tumor cells and leads to platelet activation, which promotes tumor spread. Many physiological functions of CLEC-2 have been identified by studies using CLEC-2-deficient animals. Podoplanin is not present in vascular endothelium cells, but it is expressed in lymphatic endothelial cells and a few other cells, such as kidney podocytes and type I alveolar cells. When the main lymph sac develops from the cardinal vein, podoplanin binds to CLEC-2 and activates platelets in lymphatic endothelial cells, facilitating the separation of blood and lymphatic vessels. Additionally, through homophilic contacts, CLEC-2 plays a role in thrombus stabilization under flow circumstances. Unfortunately, bleeding propensity is not markedly increased in the absence of CLEC-2. With the leading causes of death in industrialized nations being cancer and arterial thrombosis, CLEC-2 may be a potential target protein for new anti-platelet or anti-metastatic medicines with therapeutic and preventative benefits [68]. According to the literature, no studies have reported the role of s-CLE-2 in AF.

## 9. D-dimer

Elevated levels of D-dimer indicate an increase in the secondary fibrinolysis process, indicating a potential for intravascular blood clot formation. D-dimer is a substance produced during the breakdown of cross-linked fibrin and is a measurable marker of both the formation and the dissolution of blood clots. Higher D-dimer levels might indicate the presence of atrial thrombus and an increased risk of embolism in individuals with AF. D-dimer is widely recognized as a definitive test for identifying the activation of coagulation [69,70].

D-dimer has been determined to be a valuable measurement for evaluating the severity of hypercoagulability. Research has shown that individuals with heart failure have notably higher levels of D-dimer than do healthy individuals. Moreover, elevated D-dimer levels in patients with systolic heart failure serve as an independent predictor of cardiovascular mortality, even when accounting for the presence of AF [71,72].

Another study indicated that increased levels of D-dimer might serve as a distinct risk factor for the development of AF in patients with systolic HF who are hospitalized. By assessing D-dimer levels, healthcare providers can identify individuals who require more thorough monitoring following hospitalization [73,74]. Moreover, elevated levels of D-dimer can serve as an indicator of ischemic stroke in patients with acute heart failure (AHF). However, the impacts of direct oral anticoagulants on D-dimer levels have yet to be explored during hospitalization for AHF among patients with AF. A sub-analysis was conducted of a multi-center study examining the pharmacokinetics and pharmacodynamics of edoxaban in individuals with nonvalvular AF and AHF. The investigation analyzed D-dimer levels upon admission and after edoxaban initiation. D-dimer levels might be higher in patients with NVAF and AHF, particularly in those who have not received anticoagulant therapy previously. Edoxaban may be effective in reducing and maintaining the levels of D-dimer, a biomarker that predicts ischemic stroke, below the normal range in patients with NVAF and AHF [74].

The routine practice before cardioversion for non-valvular AF involves excluding left-atrial (LA) thrombus using transesophageal echocardiography (TOE). However, the use of D-dimer blood concentrations to exclude LA thrombus before cardioversion in patients with AF was investigated. This was with the aim of comparing two D-dimer cut-offs: one fixed at 500 ng/mL (DD500), which is commonly used in clinical practice; and the other adjusted to 10 times the patient’s age (DDAge), which is the cut-off used to exclude pulmonary embolism. The study found that the D-dimer cut-offs were effective in excluding LA thrombus in patients with AF. Age adjustment significantly increased the proportion of patients in whom LA thrombus could be safely excluded, thereby avoiding pre-cardioversion TOE [75].

Researchers also attempted to investigate whether there is a link between D-dimer levels and the risk of ischemic stroke in patients with NVAF, and whether high D-dimer levels could be used as a risk factor for ischemic stroke. The results showed that there was a positive correlation between D-dimer level and the risk stratification of ischemic stroke in patients with NVAF. However, D-dimer level was found to have no predictive value for the occurrence of ischemic stroke in these patients [76]. AF is a frequently occurring irregular heartbeat condition that is linked to a prothrombotic or hypercoagulable state. This condition may heighten the likelihood of cerebral and systemic embolism. The formation of most thrombi in AF patients occurs within the left atrium [77].

Tests involving D-dimer, prothrombin fragment F1 + 2, and left-ventricular ejection fraction (LVEF) can be useful in verifying hypercoagulability in patients with AF who are undergoing DOAC treatment. The results indicate that the effectiveness of apixaban as an anticoagulant might be superior to that of edoxaban in patients with NVAF, as demonstrated by lower levels of left-atrial D-dimer in the apixaban group, compared to the edoxaban group [78].

High baseline D-dimer levels were linked to a higher risk of recurrent stroke in patients with AF-related stroke and atherosclerotic diseases. This indicates that D-dimer levels could be a useful biomarker for assessing the risk of recurrent stroke in such patients. Furthermore, in patients with D-dimer levels of ≥2 μg/mL, OACs were more effective than antiplatelet therapy in reducing the risk of recurrent stroke. Therefore, using OACs for high-risk patients with AF-related stroke and atherosclerotic diseases based on D-dimer risk assessment was advisable. In terms of therapeutic strategies, it was found that there was no significant difference in the effectiveness of non-vitamin-K antagonist oral anticoagulants (NOACs) and warfarin or dual antithrombotics and OACs alone in preventing recurrent ischemic stroke in these patients. However, additional prospective randomized, controlled trials are necessary to verify these findings [79].

Prior research has shown that patients with AF exhibit elevated markers of thrombogenesis, which indicates a hypercoagulable or prothrombotic state. The impact of introducing ultra-low-dose warfarin (1 mg), conventional warfarin, or aspirin (300 mg) therapy on thrombogenesis and platelet activation in patients with AF was investigated. Compared to patients in sinus rhythm, those with AF display increased intravascular thrombogenesis and platelet activation. However, the introduction of ultra-low-dose warfarin (1 mg) or aspirin 300 mg did not produce significant changes in these markers. On the other hand, conventional warfarin therapy was found to decrease β-TG and fibrin D-dimer levels. These findings support the beneficial effects of full-dose warfarin in preventing stroke and thromboembolism in AF and suggest that ultra-low-dose warfarin and aspirin might not provide similar benefits [80].

The significance of hypercoagulability in the development of thromboembolism in AF patients is now a fact. Anticoagulation is the primary strategy for managing the prothrombotic state and preventing thromboembolic complications in this condition. D-dimer levels have been shown to reflect the varying degrees of hypercoagulability associated with AF, and they may potentially be used for monitoring and guiding treatment decisions. However, while evidence supporting the clinical utility of D-dimer is limited, future research may clarify its role in refining the thromboembolic risk assessment, in combination with clinical scores. Current studies on D-dimer in AF have primarily focused on its diagnostic and prognostic value. Despite variations in study design, the published data suggests that the D-dimer levels were associated with atrial thrombosis, predict adverse outcomes, and mortality; were linked to the volume of cerebral infarction; and could be used to assess the degree of hypercoagulability following cardioversion. Furthermore, consideration of D-dimer might potentially rule out atrial thrombus before attempting cardioversion. If larger studies confirm these findings, D-dimer testing could become a vital component of clinical decision-making for AF patients [79].

## 10. Plasminogen Activator Inhibitor-1

Inhibiting the plasminogen/plasmin system is a key function of plasminogen activator inhibitor-1 (PAI-1), which is the primary physiological inhibitor of plasminogen activators (PAs). Mainly, PAI-1 attenuates fibrinolysis because it is a tissue-type PA (tPA) fast-acting inhibitor. The role of PAI-1 extends to pericellular proteolysis, tissue remodeling, and other activities, including cell migration through suppression of urokinase-type PA (uPA) and interaction with biological ligands such as vitronectin and cell-surface receptors [81]. Among cardiac arrhythmias, AF is the most typical one. Though its exact cause is unknown, atrial fibrosis is a prominent characteristic of AF. When comparing the mRNA expression patterns of sinus rhythm and AF samples, researchers examined the *Gene Expression Omnibus* database and found 148 genes that were differently expressed. Significantly more of these genes were shown to be involved in the signaling pathway of collagen-activated tyrosine kinase receptor and the extracellular matrix assembly process. Using a protein–protein interaction network, the study was able to screen hub genes associated with atrial fibrosis. Of these hub genes, three are essential to the disease—SERPINE1/plasminogen activator inhibitor-1/PAI-1, TIMP metallopeptidase inhibitor 3/TIMP3, and decorin/DCN. Of these, PAI-1 is particularly important. A significant correlation was observed between the p53 signaling pathway and increased expression of PAI-1. Through western blot analysis, the levels of p53 and plasminogen activator inhibitor-1 protein expression were confirmed in individuals suffering from AF and sinus rhythm. In individuals with AF, the expressions of the proteins p53 and PAI-1 were shown to be elevated in the atrial tissues, when compared to the sinus rhythm controls. The results of cellular and molecular tests also revealed that p53 regulates PAI-1. As a result, the pathophysiological mechanisms of AF may include the p53/PAI-1 signaling axis, and PAI-1 may offer a novel therapeutic biomarker for AF [82].

Although it is uncertain whether tPA and PAI-1 are linked to an increased risk of stroke, they have been linked to an increased risk of incident AF. The first median values of tPA and PAI-1 were 3.1 ng/mL and 72.4 ng/mL, respectively. PAI-1 (hazard ratio [HR] 1.10, 95% Confidence interval [CI] 1.04–1.16, *p* < 0.001) and tPA (HR 1.05, 95% CI 1.01–1.08, *p* = 0.014) were linked to incidence AF in univariate analysis. However, no significant connection was detected after multivariate adjustment for body mass index, smoking, NT-proBNP, alcohol consumption, stroke, heart failure, myocardial infarction, diabetes mellitus, peripheral artery disease, and sex. The tPA and PAI-1 levels in this community-based sample were not linked to incident AF [83].

AF may often occur after heart surgery. Postoperative atrial fibrillation (POAF) might be temporary, but it can also have major side effects, including hemodynamic instability, stroke, and even mortality. In addition to acting as tissue-type plasminogen activator’s principal inhibitor, PAI-1 primarily functions as an acute-phase reactant. Increased PAI-1 alters the atrial substrate and may cause the POAF that is triggered by cardiac surgery by encouraging fibrosis and reducing extracellular matrix turnover. Researchers demonstrated a significant correlation (*p* < 0.01) between both a preoperative serum level of PAI-1 more than 15 ng/mL and a post-CPB level of PAI-1 higher than 23 ng/mL with a high incidence of POAF. Some risk factors for the development of POAF include left-atrial diameter greater than 4 cm (*p* < 0.01), advanced age (>60 years) (*p* = 0.04), history of hypertension (*p* = 0.035), number of grafts (*p* = 0.01), right coronary artery (RCA) graft (*p* < 0.01), prolonged duration of cardiopulmonary bypass (CPB) (*p* = 0.03), postoperative administration of dobutamine and epinephrine (*p* = 0.005), and a postoperative reduced ejection fraction less than 35% (*p* = 0.028). When assessed, either before or after surgery, immediately after being cut off from CPB, PAI-1 may be taken into account as a predictor of POAF [84].

Fibrosis is closely related to PAI-1, a crucial modulator of the fibrinolytic system. While PAI-1 may contribute to the development of AF and thrombosis in the elderly, it is yet unknown whether it also has a role in the development of atrial fibrosis associated with aging. Researchers investigated the relationship between PAI-1 and aging-related fibrosis by comparing the transcriptomics data of youthful (passage 4) and senescent (passage 14) human atrial fibroblasts. Electrophysiological and biochemical analyses were conducted on senescent human and mouse atrial fibroblasts, as well as those from aged animals. Ageing mice’s atrial tissue, as well as senescent human and mouse atrial fibroblasts, showed elevated expressions of the proteins p300, p53, and PAI-1. Reduced atrial fibrosis, AF inducibility, and atrial fibroblast senescence were all caused by curcumin, C646 (a p300 inhibitor), or p300 knockdown, inhibiting the production of PAI-1. Additionally, in senescent human and mouse atrial fibroblasts, p53 suppression reduced the protein expression of p21 and PAI-1. The mechanism of atrial fibrosis caused by aging may include the p300/p53/PAI-1 signaling pathway, according to research which offers new insights into the management of AF in the elderly [85].

The common promoter region variations −675G/A (4G/5G) are linked to an increased risk of thrombosis, and PAI-1, a type 1 inhibitor, controls fibrinolysis. Increased risk of cardiovascular disease has been linked to elevated levels of PAI-1. Higher levels of PAI-1 are linked to the 4G allele, which may raise the risk of intravascular thrombosis. An atrial thrombus can form in certain specific people with AF. Especially in older adults, AF quadruples the risk of stroke, increasing the risk up to five to six times. Many pathophysiological theories have been proposed to explain left-atrial thrombogenesis. Thus, the likelihood of thrombogenesis in AF may be modulated by genetic differences that impact the expression levels and activity of hemostatic factors. Researchers looked at the polymorphisms of PAI-1 4G/5G (−675G/A) in nonvalvular AF patients who had an ischemic stroke. Age and gender differences were not statistically significant across the groups. The genotype distribution of the groups did not vary statistically. The group of NVAF patients who suffered a stroke had the following genetic distribution: the frequency of the 5G/5G, 5G/4G, and 4G/4G genotypes was 29 (41.4%), 27 (38.6%), and 14 (20%), respectively. In the control group, the genotype distribution was as follows: the frequency of the 5G/5G genotype was 37 (52.9%), the 4G/5G genotype was 23 (32.9%), and the 4G/4G genotype was 10 (14.3%). The genotype distributions did not differ across the groups in a statistically meaningful way. According to these findings, nonvalvular AF and ischemic stroke in the Turkish population do not appear to be related to the 4G/5G polymorphism of the PAI-1 gene [86].

Enzyme-linked immunoassays and colorimetric assays were utilized to evaluate the levels of plasminogen, tPA, PAI-1, α2-antiplasmin activity (α2-AP), D-dimer, and vitronectin. After the beginning of PAF, patients were admitted to the hospital between the 2 h and the 24 h marks (mean 8.14 ± 0.76 h). In comparison to the control group, the patient group exhibited substantially higher levels of plasminogen (159.40 ± 4.81 vs. 100.2 ± 2.88%, *p* < 0.001) and tPA (11.25 ± 0.35 vs. 6.05 ± 0.31 ng/mL, *p* < 0.001). In comparison to the 15.15 ± 0.52 AU/mL group, the PAF group had lower levels of PAI-1 activity (7.33 ± 0.37) and α2-AP (112.9 ± 2.80 vs. 125.60 ± 3.74%, *p* < 0.05), as well as lower levels of vitronectin plasma (134.7 ± 5.83 vs. 287.3 ± 10.44 mcg/mL, *p* < 0.001). In contrast, patients, as compared to the control group, had much higher D-dimer levels (0.53 ± 0.07 vs. 0.33 ± 0.02 ng/mL, *p* < 0.05). PAF is characterized by early alterations in the fibrinolytic system, which may have a direct bearing on the disease’s presentation. In the initial 24 h of the illness, there is elevated plasma fibrinolytic activity, which is probably a pathophysiological reaction to the accelerated procoagulation processes [87].

## 11. Platelet Activation

The smallest component of blood, platelets play a crucial role in hemostasis and thrombosis. To establish hemostasis, initial platelet adhesion, activation, and aggregation following tissue damage activate coagulation factors and other mediators. Furthermore, these well-coordinated actions are essential biological processes that favor wound healing after tissue injury. Since its inception one century ago, the study of platelet function has gained importance in the monitoring of bleeding, antiplatelet activity, platelet dysfunctions, and coagulation factors. To lessen the hematological problems, it is very helpful to comprehend the platelet thrombogenicity cascade [88].

In hemostasis and thrombosis, platelet activation and blood coagulation are complimentary, mutually reliant processes. Many coagulation factors interact with platelets, and thrombin, a coagulation product, is a strong agonist that activates platelets. These actions are supported by platelet-bound factor Va and factor IXa. Moreover, by acting as prothrombin and factor XI binding sites, platelets can aid in the starting phase of coagulation. As a result, they assume the role of tissue-factor and factor VIIa in the initialization of coagulation [89].

There is limited information regarding the relationship between thrombocytopenia, or low platelet count, and mortality in patients with AF who are on oral anticoagulant therapy. The factors that contribute to thrombocytopenia in a large group of AF patients and whether thrombocytopenia was associated with overall mortality were examined. It was suggested that thrombocytopenia is a common occurrence in AF patients who are taking oral anticoagulants, and despite an increased risk of mortality, thrombocytopenia might not necessarily be a direct risk factor for mortality. Instead, it could be a reflection of the presence of comorbidities that were linked to poor survival [90].

Platelet function in AF patients, specifically, exploring how it relates to abnormal thrombogenesis, was investigated. The study was focused on platelet activation and the effects of the concomitant use of anticoagulant and antiplatelet therapies. It was shown that patients with AF exhibit changes in plasma markers of platelet function, but no significant platelet aggregation dysfunction. However, treatment with warfarin or aspirin did not show significant favorable effects on platelet activation, even though warfarin use was linked with diminished thrombogenesis (fibrin D-dimer). Accordingly, Kamath et al. suggested that platelet activation is not a reliable biomarker in the development of thromboembolism in AF [66].

Whether paroxysmal atrial fibrillation (PAF) is a risk factor for stroke, possibly similar to chronic AF (CAF), and whether it affects the thrombotic state by increasing mean platelet volume (MPV) was examined. MPV reflects the size of the platelet, and its activation and function. High MPV levels indicate larger and more active platelets that release higher levels of thromboxane A2 compared to smaller platelets. The results showed that inflammatory markers such as CRP and erythrocyte sedimentation rate and MPV, which is a marker of platelet size and activity, were higher in patients with PAF. It was suggested that PAF might contribute to a prothrombotic state [91].

Patients with chronic AF have an increased risk of stroke due to a hypercoagulable state, but it is unclear whether this state is caused by AF alone or the underlying disease. There is a lack of information on fibrin coagulation properties in patients with PAF. To examine if AF alone influences the fibrin coagulation system, the relationship between fibrin coagulation activity and duration of AF in PAF patients was investigated. It was found that AF alone forces platelet aggregation and coagulation, and that both were influenced by the duration of the AF. The enhancement of platelet activity and coagulability happens 12 h after the onset of AF [6]. Also, a decrease in platelet count (PLT) was linked to a reduced risk of stroke, but an increased risk of bleeding events. When combined with the traditional risk factors, the use of PLT improved the prediction of stroke [92].

The risk of bleeding in anticoagulated patients can increase due to a reduction in either platelet count or function. Studies imply that low PLT can increase the risk of bleeding in NVAF patients who are taking dabigatran after catheter ablation [93].

MPV has been linked to an increased risk of stroke in patients with AF, but it is unclear whether, in patients with NVAF, it can predict left-atrial stasis, which is assessed through transesophageal echocardiography. These results indicate that MPV might be connected to the existence of indicators of left-atrial stasis, which supports the idea that the association between mean platelet volume and stroke in patients with NVAF was likely due to a cardioembolic mechanism [94].

Researchers attempted to evaluate the safety and effectiveness of left-atrial appendage occlusion (LAAO) in patients with AF and chronic thrombocytopenia (cTCP) over the long term. LAAO using the Watchman device may be a viable option for preventing stroke in patients with AF, regardless of whether they have thrombocytopenia. However, it is important to closely monitor cTCP patients for potential bleeding complications [95]. The risk of blood clotting is a major complication of AF. Platelets and microparticles (MPs) play important roles, both in blood clotting and in the stopping of it, but their roles during AF are not well understood. The findings suggest that an acute episode of AF can lead to a decrease in MPs-associated tissue-factor activity, which may result from consumption, favoring coagulation and local production of thrombin. Reduced platelet basal aggregation to the thrombin receptor activating peptide (TRAP) may be caused by platelet activator receptor 1 (PAR1) desensitization, whereas improved response after an induced episode of AF suggests activation of coagulation and PAR1 re-sensitization [96].

Patients with AF are at an increased risk for cognitive impairment, possibly due to a hypercoagulable state and an immune inflammatory response. Platelets can contribute to immune inflammation, but it is unclear whether platelet count is related to cognitive function in these patients. A nonlinear relationship was found between platelet count and cognitive function in patients with AF, after adjusting for confounding factors. The optimal platelet count for preserving cognitive function in these patients was found to be around 230, and platelet counts that were too high or too low may have had negative effects on cognitive function [97].

The primary focus in managing AF is the prevention of thromboembolism, as AF itself is a separate risk factor for both thromboembolism and stroke. The risk of these events is amplified approximately three to five times in individuals with AF. Therefore, prioritizing measures to prevent thromboembolism is crucial in the management of AF [98].

AF predisposes individuals to the formation of blood clots in the heart’s chambers. These clots can then dislodge and travel to the brain, causing a stroke. Coagulation factors and markers, such as PT, aPTT, and INR, are used to assess the blood’s clotting ability and help estimate the risk of stroke in AF patients. AF patients with a moderate to high risk of stroke are often prescribed anticoagulant medications to reduce the formation of blood clots. Coagulation factors and markers, particularly the INR, are used to monitor the effectiveness of anticoagulation therapy, such as that with warfarin. Regular monitoring helps ensure that the patient’s blood is within the desired therapeutic range, balancing the prevention of clotting with the avoidance of excessive bleeding. Coagulation factors and markers can provide valuable information about an individual’s coagulation profile, which can vary among patients. By assessing these factors, healthcare professionals can tailor the treatment approach for each patient. For instance, some AF patients may have underlying conditions, such as liver disease, that affect their coagulation status, which may necessitate adjustments in anticoagulation therapy.

Because of the related thromboembolic effects, AF is an “epidemic” condition associated with significant risks of morbidity and death. Even after much research, thrombogenesis in AF is still a complicated issue. The basis of thrombogenesis, which is defined by abnormalities in blood flow, vascular wall, and blood components, appears to be Virchow’s triad, which was posited 150 years ago. Platelets and the endothelium are among the blood elements that exhibit aberrant hemostasis, which might contribute differently to a hypercoagulable condition. Antiplatelet medication is still a common treatment for AF; however, its effectiveness is questionable in those without related risk factors and appears to be minor in those who have them [99]. 

Prothrombotic states are linked to AF, according to an increasing body of research. Hemostasis indicators’ function in AF is still unclear, nonetheless. AF cases had significantly higher levels of circulating platelet factor-4, β-thromboglobulin (BTG), and P-selectin when compared to controls in terms of platelet activation, respectively, as to coagulation activation; increased levels of plasma D-dimer, fibrinogen, thrombin-antithrombin, prothrombin fragment 1 + 2, and antithrombin-III were significantly associated with AF. When AF cases were compared to controls, tissue-type plasminogen activator and PAI-1 showed significantly higher levels of fibrinolytic function, respectively. However, these associations were no longer significant when subgroup analysis based on anticoagulant treatment status was conducted. As for endothelial function, there was no correlation found for soluble thrombomodulin, while a higher vWF was substantially linked with AF. The following circulating hemostatic factors have a substantial correlation with AF: PF-4, BTG, P-selectin, D-dimer, fibrinogen, TAT, F1 + 2, AT-III, and vWF. The exact mechanism of the prothrombotic condition and how hemostatic indicators facilitate thromboembolism in AF require more investigation [100].

A total of forty-five individuals with nonvalvular AF who had not taken anticoagulants were assessed by transesophageal echocardiography to examine the connection between hemostatic indicators and left-atrial (LA) flow dynamics. LA spontaneous echo contrast (SEC) or thrombus was detected, and the flow of the appendages was ascertained. Utilizing peripheral blood as a hemostatic marker, we assessed the plasma levels of beta-thromboglobulin, fibrinopeptide A, D-dimer, thrombin-antithrombin III complex (TAT), and platelet factor 4. Of the patients in the low-velocity group, 11 (58%) had LA SEC or thrombus, whereas none of the patients in the high-velocity group demonstrated either (*p* < 0.001). In comparison to the high-velocity group, the low-velocity group had considerably greater plasma levels of TAT, fibrinopeptide A, D-dimer, beta-thromboglobulin, and platelet factor 4. Compared to twenty-six patients in the high-velocity group, eight patients in the low-velocity group did not have LA SEC or thrombus, and their plasma levels of TAT, fibrinopeptide A, beta-thromboglobulin, and platelet factor 4 were considerably greater. Intravascular coagulation–fibrinolysis activity and platelet activation were elevated in patients with nonvalvular AF who also had a larger and dysfunctional LA or a lower LA appendage flow velocity. In individuals with nonvalvular AF, these anomalies could be intimately associated with their thrombogenic condition [101].

In comparison to the healthy controls, both nonvalvular AF groups exhibited significantly higher concentrations of vWF, factor VIII: C, fibrinogen, D-dimer (a fibrinolytic product), beta-thromboglobulin, and platelet factor 4. They also exhibited significantly higher fibrinogen/antithrombin ratios and significantly higher spontaneous amidolytic activity. Both groups with NVAF had considerably reduced prekallikrein levels. When compared to the healthy controls, stroke patients with sinus rhythm exhibited normal hemostatic function, normal quantities of platelet-related components, and a slightly elevated concentration of fibrinopeptide A. The two groups with nonvalvular AF differed from the healthy controls, as they were composed of stroke patients with a sinus rhythm. Patients with nonvalvular AF who had previously experienced an ischemic stroke did not exhibit different hemostatic functions. Therefore, individuals with nonvalvular AF may be at higher risk for stroke due to changes in their hemostatic function [102].

Multiple co-morbidities and anticoagulants following TAVR are common in patients with AF, which contributes to a poor prognosis, including bleeding episodes. A vWF high molecular weight (HMW) multimers deficiency can be surrogate-detected by the major hemostasis point-of-care test known as closure time adenosine diphosphate (CT-ADP). Early and late major/life-threatening bleeding complications (MLBCs) appear to be significantly influenced by the duration of CT-ADP (>180 s) during TAVR. Bleeding problems were evaluated according to the VARC-2 (Valve Academic Research Consortium-2) standards. In a study following patients within the year following TAVR, a composite of all-cause mortality, myocardial infarction, stroke, and hospitalization for heart failure was considered a major adverse cardiac and cerebrovascular event (MACCE). With post-procedure CT-ADP > 180 s, an ongoing main hemostasis problem was identified. One-year MACCE was the secondary goal, whereas the primary endpoint was the incidence of MLBCs in the first year. In comparison with non-AF patients, patients with AF had higher incidences of all-cause mortality (15% vs. 8%, *p* = 0.002), MACCE (29% vs. 20%, *p* = 0.002), and MLBCs (20% vs. 12%, *p* = 0.001), after one year. When the sample was divided into four categories based on AF and CT-ADP >180 s, the patients with these conditions had the highest likelihood of MLBCs (log-rank test; *p* < 0.001). MLBCs within a year were shown to be 4.6 times more common in patients with AF and CT-ADP >180 s than in non-AF individuals with CT-ADP ≤ 180 s, according to a multivariate Cox regression analysis (hazard ratio: 4.60; 95% confidence interval: 2.18–9.68; *p* < 0.001). MLBCs at a 1-year follow-up were shown to be strongly independently predicted in TAVR patients by AF with post-procedural CT-ADP > 180 s. A customized and risk-adjusted antithrombotic treatment following TAVR may be considered for patients with persistent primary hemostasis abnormalities, since it indicates that these conditions increase the risk of bleeding events, especially in patients with AF [103].

Perhaps as a result of promoting cardioembolism and a prothrombotic condition, AF is linked to dementia and cognitive impairment. When comparing people with AF and dementia to those without dementia, a study discovered indications of enhanced thrombin production and fibrin turnover. When using warfarin for an extended period, individuals with AF may be protected from developing dementia [104].

Another study provided a summary of warfarin’s anticoagulant impact and emphasized the benefits of DOACs for those with NVAF. However, it is challenging to use in large-scale clinical applications because of the absence of adequate monitoring, the fact that they cost more than warfarin, and the fact that some anticoagulant antagonists are not even available in certain regions. As for DOACs, the principal benefits are as follows: lower risk of cerebral bleeding without the need for regular monitoring, predictable pharmacokinetics, high effectiveness, short half-life and quick elimination of the effect after withdrawal, and reduced requirements for pharmacological and dietary limits. Utilizing DOACs can help patients better adhere to long-term anticoagulant medication, which will raise the rate at which AF treatment is successful [105].

Individuals with NVAF (i.e., no prosthetic valves or rheumatic mitral valve disease) seem to benefit more from the newer, non-vitamin-K-dependent oral anticoagulants, such as direct thrombin or Xa inhibitors, in terms of safety and efficacy in avoiding thromboembolism, as compared to warfarin [106].

An increased risk of stroke occurs with AF, which is one of the world’s major causes of mortality and disability. Oral anticoagulation improves outcomes for AF patients who are considered by recognized criteria to be at moderate or high risk of stroke. Appropriate patient selection is necessary, nevertheless, to guarantee that the advantages outweigh the bleeding hazards. The cornerstone of therapy has been vitamin-K-antagonistic medicines. However, there are other more recent medications with unique mechanisms. In the large-scale randomized studies of individuals with NVAF, two oral anticoagulants—direct thrombin inhibitors and factor Xa inhibitors—have shown their safety and effectiveness, while circumventing many of the drawbacks of warfarin. Nonetheless, there is still a clinical problem in managing patients on warfarin or new medications. When choosing anticoagulant medication, patients with AF should take into account several crucial factors [107].

## 12. Conclusions

Fibrinogen and fibrin, prothrombin, thrombomodulin, soluble urokinase plasminogen activator receptor, von Willebrand factor, P-selectin, D-dimer, plasminogen activator inhibitor-1, and platelet activation play significant roles in the pathogenesis of AF. However, many other coagulation markers and factors have not been studied yet.
